# Galectin 3 and Galectin 3 Binding Protein Improve the Risk Stratification after Myocardial Infarction

**DOI:** 10.3390/jcm8050570

**Published:** 2019-04-26

**Authors:** Giulia Gagno, Laura Padoan, Elisabetta Stenner, Alessandro Beleù, Fabiana Ziberna, Cristina Hiche, Alessia Paldino, Giulia Barbati, Gianni Biolo, Nicola Fiotti, Tarcisio Not, Antonio Paolo Beltrami, Gianfranco Sinagra, Aneta Aleksova

**Affiliations:** 1 Cardiovascular Department, Azienda Sanitaria Universitaria di Trieste and Department of Medical Surgical and Health Sciences, University of Trieste, 34129 Trieste, Italy; giulia.gagno@studenti.units.it (G.G.); cristina.hiche@asuits.sanita.fvg.it (C.H.); alessiapaldino@gmail.com (A.P.); gianfranco.sinagra@asuits.sanita.fvg.it (G.S.); 2 Sport and Exercise Medicine Division, Department of Medicine, University of Padova, 35122 Padova, Italy; argonauta92@hotmail.it; 3 Unique Laboratory of ASUITs, Burlo, Gorizia and Monfalcone, Azienda Sanitaria Universitaria di Trieste, 34100 Trieste, Italy; elisabetta.stenner@asuits.sanita.fvg.it; 4 Department of Radiology, G.B. Rossi Hospital, University of Verona, 37134 Verona, Italy; ale.beleu@gmail.com; 5 Institute for Maternal and Child Health–IRCCS “Burlo Garofolo” Trieste and University of Trieste, 34100 Trieste, Italy; fabyz1@alice.it (F.Z.); tnot@units.it (T.N.); 6 Biostatistics Unit, Department of Medical Surgical and Health Sciences, University of Trieste, 34100 Trieste, Italy; giulia_barbati@yahoo.it; 7 Unit of Clinica Medica Generale e Terapia Medica, Department of Medical Surgical and Health Sciences, University of Trieste, 34100 Trieste, Italy; biolo@units.it (G.B.); fiotti@units.it (N.F.); 8 Department of Medicine (DAME), University of Udine, 33100 Udine, Italy; antonio.beltrami@uniud.it

**Keywords:** Galectin 3, Galectin 3 binding protein, myocardial infarction, ischemic cardiomyopathy, outcome, mortality, reinfarction, angina, recurrent ischemic events

## Abstract

Background: Acute myocardial infarction (AMI) survivors are at risk of major adverse cardiac events and their risk stratification is a prerequisite to tailored therapeutic approaches. Biomarkers could be of great utility in this setting. Methods: We sought to evaluate the utility of the combined assessment of Galectin 3 (Gal-3) and Galectin 3 binding protein (Gal-3bp) for post-AMI risk stratification in a large, consecutive population of AMI patients. The primary outcomes were: Recurrent angina/AMI and all-cause mortality at 12 months after the index event. Results: In total, 469 patients were included. The median Gal-3bp was 9.1 μg/mL (IQR 5.8–13.5 μg/mL), while median Gal-3 was 9.8 ng/mL (IQR 7.8–12.8 ng/mL). During the 12 month follow-up, 34 patients died and 41 had angina pectoris/reinfarction. Gal-3 was associated with all-cause mortality, while Gal-3bp correlated with the risk of angina/myocardial infarction even when corrected for other significant covariates. The final multivariable model for mortality prediction included patients’ age, left ventricular ejection fraction (LVEF), Gal-3, and renal function. The ROC curve estimated for this model has an area under the curve (AUC) of 0.84 (95%CI 0.78–0.9), which was similar to the area under the ROC curve obtained using the GRACE score 1-year mortality. Conclusions: The integrated assessment of Gal-3 and Gal-3bp could be helpful in risk stratification after AMI.

## 1. Introduction

Coronary heart disease is still the leading cause of death worldwide [1]. Nevertheless, with the adoption of coronary revascularization therapy and secondary prevention measures, the survival rates after acute ischemic events have increased, and, therefore, the prevalence of patients surviving acute myocardial infarction (AMI) is growing [2]. Patients that experienced an acute ischemic coronary event are at risk of major adverse cardiac events, such as cardiac death or reinfarction, which is a strong independent predictor of mortality [3].

Risk stratification of AMI patients is a prerequisite to optimize patient-tailored therapeutic approaches in order to improve prognosis.

To fulfil this task, biomarkers could be of great utility, since they could provide objective information that is easily understood. Single biomarkers as well as different combinations of biomarkers have been proven to be of some utility in defining prognosis in selected patients affected either by ST-elevation myocardial infarction-STEMI [4] or non ST-elevation myocardial infarction-NSTEMI [5,6,7,8,9]. 

Data on the usefulness of the integrated use of Galectin 3 and Galectin 3 binding protein in the setting of acute myocardial infarction are lacking. Galectin 3, a member of the galectin family, is increased in the left ventricle in the early post-ischemic period [10] that follows AMI and its serum levels are proven to correlate with the myocardial infarct size (quantified using cardiac magnetic resonance) [11] and with adverse ventricular remodeling and heart failure [7,12]. Galectin 3 binding protein (also known as Mac-2 binding protein, M2BP) is a highly glycosylated secreted protein widely expressed in most normal tissues and body fluids [13]. However, an elevation of galectin-3 binding protein levels was observed in patients with different cancer histotypes, with autoimmune and infectious diseases, such as hepatitis C, bronchial asthma, or human immunodeficiency virus infection. Recently, Galectin 3 binding protein levels have been proposed to play an additional role in cardiovascular disease, specifically, in the development and destabilization of atherosclerotic plaques [13,14]. 

In this work, we aimed to determine if Galectin 3 and Galectin 3 binding protein tested together could be useful for event prediction in a large, unselected consecutive population of patients affected by myocardial infarction.

## 2. Material and Methods

### 2.1. Study Population

We prospectively enrolled 469 consecutive patients, either admitted or transferred to the Cardiovascular Department of the University Hospital in Trieste with a diagnosis of acute myocardial infarction (both STEMI and NSTEMI). The diagnosis was established by the cardiologist of the intensive cardiac care unit, according to ESC guidelines [15,16]. Every patient underwent: a coronary angiography within the first 24 h after symptoms onset; a detailed clinical exam, including an electrocardiogram (EKG); a transthoracic echocardiogram; and standard laboratory analyses. Patients were carefully and continuously monitored for the full length of their hospitalization until they were either discharged or moved to another department/hospital. Subsequently, patients were subjected to a clinical and echocardiographic follow-up.

The study protocol was designed according to the ethical guidelines outlined in the Declaration of Helsinki and was approved by our local ethic committee (N° 67/2015). All patients provided written informed consent. Inclusion criteria were: Age > 18 years, myocardial infarction with clinical onset in the previous 24 h, and written informed consent for study participation. We excluded patients with active malignancy with a poor prognosis (life expectancy less than 12 months) and those with an inability to understand the nature and purpose of the study.

For each enrolled patient, demographic characteristics, past and family history, medical treatment at admission and discharge, baseline characteristics, clinical data during hospital stay and at discharge, performed medical procedures, hospital course, and outcome were entered into a database.

Data on patients’ follow-up were obtained by consulting the electronic health records software, Cardionet (INSIEL, Trieste, Italy) and G2 Clinical (INSIEL, Trieste, Italy). The data for those who came from other provinces (Udine, Gorizia, Monfalcone, and Palmanova), were obtained thanks to the collaboration with the referring hospitals.

The end of the follow-up was either fixed at 12 months after the index event or on the date of death of the patient, in order to record events that occurred in the first 12 months.

### 2.2. Definitions, Endpoints, and Follow-Up

The primary outcomes were recurrent angina or myocardial infarction and all-cause mortality at 12 months after the index event. 

Angina and myocardial infarction were diagnosed according to ESC guidelines [15,16] and the third definition of myocardial infarction, respectively [17].

Multistage revascularization (initial revascularization of the culprit lesion only, followed by revascularization of ≥1 lesions as a planned procedure at a later date) was not considered as an end-point.

For the event mortality, our multivariable model was compared to the GRACE score, which was calculated using the official calculator (www.gracescore.org). The GRACE score for 1 year mortality was the one used in our analysis. 

Multi-vessel critical coronary artery disease was defined as the presence of >70% stenosis in at least two coronary vessels and detected by coronary angiography.

### 2.3. Measurements of Biomarkers

Venous blood samples were collected in ethylenediaminetetraacetic (EDTA) tubes within 8 h from admission, blood samples were then processed in order to obtain plasma (centrifuged at 2500 *g* for 10’ at 4 °C), which was stored at −80 °C until analysis was performed.

Plasma levels of Galectin 3 binding protein were measured using the competitive ELISA immunoassay Human s90K Mac-2 binding protein Paltinum (Life Technologies, Thermofisher, US) according to the manufacturer’s instructions. Minimum detectable concentration of Galectin 3 binding protein was 0.012 µg/mL with an intra-assay coefficient of variation (CV) of 5.5%, an inter-assay CV of 11.9%, and a sensitivity of 0.00092 µg/mL.

The plasma concentrations of Galectin 3 were measured using commercially available sandwich ELISA kit, Galectin 3 (R&D Systems Europe, Abingdon, UK), following the manufacturer’s instructions. Inter-assay and intra-assay coefficients of variability were 6.2% and 3.7% (Galectin 3), respectively. The sensitivity of the galectin test was 0.016 ng/mL.

For the determination of plasma levels of IL-1β, a high-sensitivity ELISA kit (Quantikine HS Human IL-1β Immunoassay, R&D Systems, Inc., Minneapolis, MN, USA) was used. The kit range was 0.1 to 8 pg/mL. The mean minimum detectable dose was 0.063 pg/mL. IL-1β concentration was measured by subtracting the 540 nm from the 450 nm spectrophotometer readings.

### 2.4. Statistical Analysis

Continuous variables are presented as mean values (with the standard deviation) and as median values (with the interquartile range) when departing from the normality assumption, verified by means of the Kolmogorov–Smirnov test. Categorical variables are presented as proportions.

Characteristics—measured as continuous variables—of different groups of patients were compared respectively using ANOVA or Mann–Whitney tests. Categorical variables were compared using the Chi-square test of the Fisher exact test if necessary.

Linear association between parameters was investigated using the Pearson correlation coefficient or Spearman Rho, accordingly to the distribution.

The cumulative probability of events during the follow-up was estimated using the Kaplan–Meier method. The cumulative incidence of angina and reinfarction estimated by the Kaplan–Meier method was then compared with the cumulative incidence of this event considering death as a competing risk, which was estimated using the R library “cmprsk” that implements the method described by Gray et al. [18]. Cause-specific Cox proportional hazard models were used to assess univariable predictors of the events of angina and reinfarction, censoring deaths, and standard Cox model for all-cause mortality. Levels of biomarkers were entered in the Cox analysis as continuous variables of the original variable or after logarithmic transformation. The multivariable Cox models included variables significant at the univariable Cox regression and clinically relevant for the specific event.

This model was then reduced by means of a backward conditional stepwise procedure. 

Receiver-operating characteristics (ROC) analysis was performed to assess the performance of the final multivariable model to predict all-cause mortality. Area under the ROC curves of our model was compared to the area under the ROC of the GRACE score for 1 year mortality using DeLong test. 

Classification and regression tree (CRT) analysis, a non-parametric binary recursive partitioning method, was employed to predict all-cause mortality using the list of variables selected by the multivariable model [19,20]. This approach had already been used in other studies to improve upon performance of single biomarkers for distinguishing between different end-points [21]. All the variables entered in the model were continuous variables and the biomarker level was forced to be the primary variable in the model. The minimum number of cases was designated as 10 for parent nodes (prior to split) and 5 for child nodes. In our case, the specific interest was in exploring a “data-driven” cut-off for the variables of interest in splitting our population. 

All statistical tests were two-sided, and statistical significance was set at *p <* 0.05. Statistical analyses were performed using the software, IBM SPSS Statistical Package for Mac, version 19, and the R statistical software, version 3.4.0. 

## 3. Results

### 3.1. Study Population 

The study cohort (Table 1) consisted of 469 consecutive patients with acute myocardial infarction. In the cohort, 60% of them presented with ST-segment elevation acute myocardial infarction. Mean age at admission was 67.6 (SD 11.3) years and about 68% were male. A history of previous myocardial infarction or coronary revascularization (percutaneous coronary intervention or coronary artery bypass grafting) was present in 19% of patients. Seventy percent of patients were treated with percutaneous coronary intervention (a drug-eluting stent (DES) was implanted in 73.7% of patients). At pre-discharge evaluation, the majority of patients was asymptomatic in NYHA class I, with median left ventricular ejection fraction of 54% (IQR, 47–60%).

The median Galectin 3 binding protein plasma level was 9.1 µg/mL (IQR, 5.8–13.5 µg/mL) and strongly correlated with critical multivessel coronary artery disease (*r* = 0.13, *p* = 0.006), with BMI (*r* = 0.18, *p <* 0.001), as well as with other markers of inflammation: IL-1β (*r* = 0.1; *p* = 0.03), hsCRP (*r* = 0.24; *p <* 0.001), and fibrinogen (*r* = 0.15; *p* = 0.003). The median value of Galectin 3 was 9.8 ng/mL (IQR 7.8–12.8 ng/mL). Patients with Galectin 3 higher than the median value were significantly older and had a significantly higher white blood cell count, when compared with patients with Galectin 3 levels under the median value. Galectin 3 correlated with white blood cell count (*r* = 0.15, *p* = 0.002) and with the markers of inflammation, such as IL-1β (*r* = 0.16; *p* = 0.012), hsCRP (*r* = 0.16; *p <* 0.001), and fibrinogen (*r* = 0.15, *p* = 0.002). No correlations were observed between Galectin 3 levels and mononuclear cell counts (*r* = −0.2, *p* = 0.67).

### 3.2. Outcome During the Follow-Up

During the follow-up period of 12 months, 75 patients reached the primary outcomes: 34 deaths (7.2%) and 41 angina pectoris requiring hospitalization/revascularization or myocardial infarction (8.7%). Fourteen patients died within the first month. Thus, they were excluded from the analyses for the event angina or reinfarction. A summary of the characteristics of the entire population of patients (*n* = 469) and of the cohort of patients analyzed for the angina event (*n* = 455) is provided in Table 1 and in Supplementary Table S1, respectively. The main characteristics of the patients, stratified by endpoints, are shown in Table 2a,b.

#### 3.2.1. Angina and Reinfarction during Follow-Up

**Figure 1:**
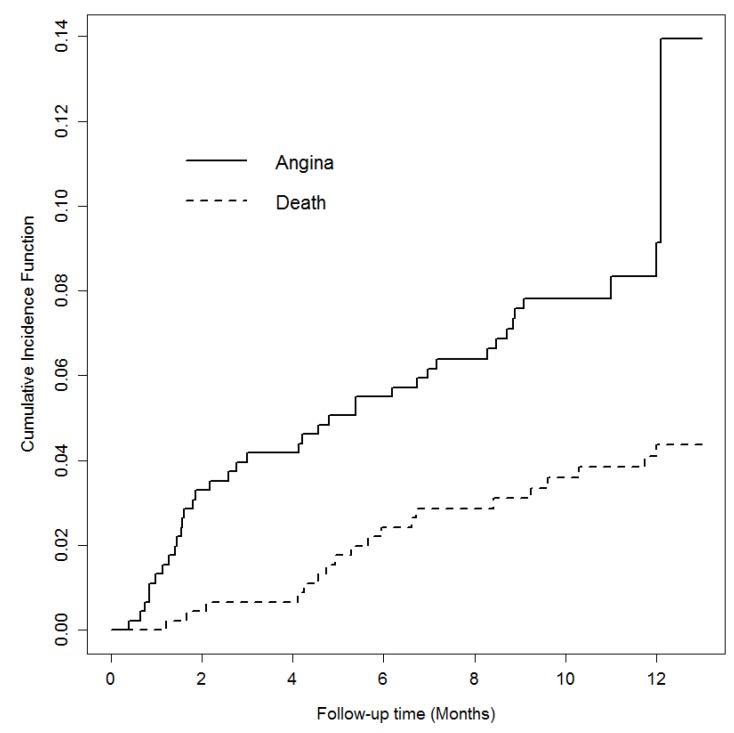
Curves of the cumulative incidence of events.

The Kaplan–Meier estimated cumulative rate of angina pectoris requiring hospitalization/revascularization or reinfarction during the follow-up was 9% and the event occurred a median of 4.2 (1.5–8.6) months after the index event. When we considered the competing risk of death, the cumulative incidence of angina or reinfarction during the first 12 months remained the same at 9% (Figure 1). Among the patients with angina that needed revascularization, only two of them had a restenosis of the culprit lesion.

Univariable cause-specific Cox regression analysis revealed that male gender, STEMI diagnosis, diabetes mellitus, previous acute myocardial infarction, percutaneous coronary intervention or coronary artery bypass grafting, hemoglobin level at discharge, and Galectin 3 binding protein level were associated with an increased risk of angina/reinfarction (Supplementary Table S2). Next, we performed a multivariable analysis including all the significant covariates of the univariable analysis together with the relevant covariates proposed in a recent study by Arnold et al. [22] (socio-economic variables, such as medical insurance and level of employment, were excluded since in our country, we have a public healthcare system). After a backward stepwise variable selection, the following clinical variables were identified as independent predictors of angina/reinfarction (Table 3a): Male gender, STEMI diagnosis, and diabetes mellitus. However, with regard to the biomarkers, only plasma Galectin 3 binding protein levels were significantly associated with the risk of developing another ischemic event during the first 12 months following the acute myocardial infarction (Table 3a).

#### 3.2.2. Mortality during Follow-Up

The Kaplan–Meier estimated cumulative rate of death was 7.5% and the event occurred a median of 4.1 (0.4–6.7) months after the index event.

Patients who died during the follow-up had a significantly higher median value of Galectin 3 at baseline (*p* < 0.001) (Table 2b), while the median Galectin 3 binding protein value was not significantly different in the two groups of patients (Table 2b). In the multivariable Cox regression analysis, after adjusting for other variables that were significant in the univariable analysis (Supplementary Table S3) and clinically relevant, LnGalectin 3 levels were significantly associated with the risk of death. The final multivariable model included patients’ age, left ventricular ejection fraction, LnGalectin 3, and renal function at discharge, as shown in Table 3b.

**Figure 2:**
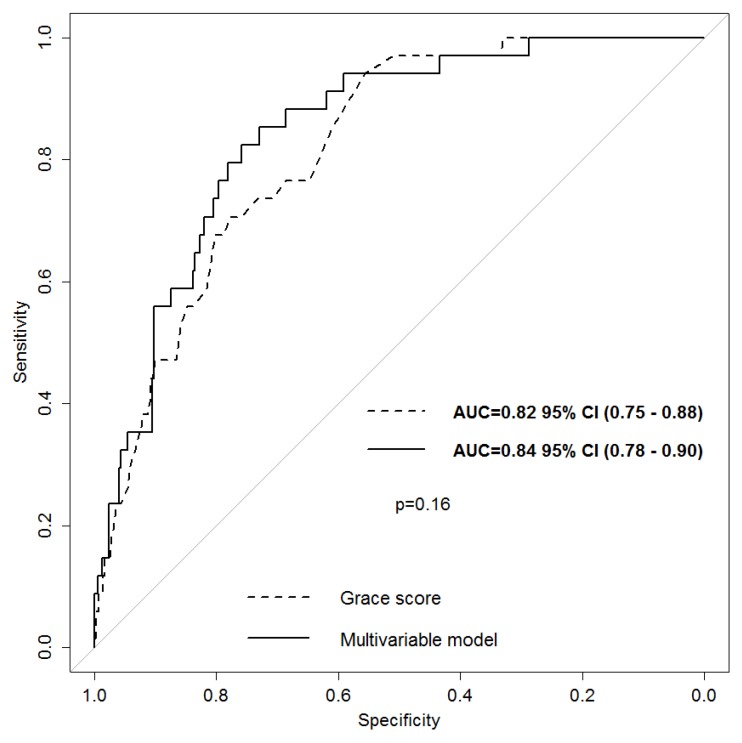
ROC curve of our multivariate model compared to the ROC curve of the GRACE score for mortality at 1 year.

Receiver-operating characteristics (ROC) curve was estimated to assess the performance of the final multivariable model to predict one-year all-cause mortality. The area under the curve (AUC) was 0.84 (95% CI 0.78–0.90), which was similar to the area under the ROC curve obtained using the GRACE score 1-year mortality (AUC = 0.82, 95% CI 0.75–0.88), De Long Test, *p* = 0.16, Figure 2. 

In order to verify the “optimal” cut-off of the variables from our final multivariable model to classify mortality in patients with acute myocardial infarction, a classification and regression tree was generated. Variables entered in the model were: Galectin 3 after logarithmic transformation, patients’ age, left ventricular ejection fraction, and renal function at discharge. The minimum number of cases was designated as 10 for parent nodes (prior to split) and 5 for child nodes. The variables which were maintained in the classification tree were LnGalectin 3, patients’ age, and left ventricular ejection fraction (which, of note, were the ones with *p* < 0.05 at the multivariable analysis). The model we obtained identified the end-point of survival at one year with 100% specificity and 93% of the negative predictive value (VPN) while its utility in predicting mortality was very low, therefore, it proved to be a good rule out model for identifying patients with a good outcome at the one-year follow-up. The cut off of LnGalectin 3 identified by the model was 2.285 on the log scale, which corresponds to the value 9.82 of the original variable, as shown in Supplementary Figure S1.

## 4. Discussion

In this work, we proved for the first time, in a large, unselected, consecutive, real world population of patients with myocardial infarction, that the dosage of Galectin 3 and Galectin 3 binding protein, within the first 24 h after the acute event, could help to better stratify patients’ risk and to provide additional and integrated information about the probability of distinct events. Specifically, Galectin 3 was an independent predictor of all-cause mortality, while Galectin 3 binding protein levels were independently associated with a higher risk of angina pectoris or reinfarction. Importantly, the characteristics of the population enrolled in our study are in accordance with what emerges from international [2,23,24] and national epidemiological studies [25]. Also, the rates of both one year mortality and one year angina/reinfarction observed in our cohort of patients are in line with those reported by the literature [22,26]. 

This association with distinct events may relate to the fact that each biomarker reflects different pathobiological axes of the post myocardial infarction response [4,5].

Specifically, Galectin 3 binding protein is a highly glycosylated protein, expressed in most normal tissues and body fluids [13], that seems to be involved in different physiological processes, such as cell adhesion and immunomodulation [13,14]. Even if data on the pathophysiological role played by Galectin 3 binding protein in cardiovascular disease are still lacking, it has been suggested that its levels correlate with a poor cardiovascular outcome [13]. Concerning our population study, we observed that the levels of Galectin 3 binding protein detected in our population were higher than those detected in both healthy individuals [8,27], and in patients with stable coronary disease [14]. This finding is in line with the knowledge that if Galectin 3 binding protein has a role in the inflammatory process which leads to progression and destabilization of atherosclerotic plaques [13], it should be highly expressed in patients who suffered acute myocardial infarction, which in most cases is due to a thrombotic occlusion of a coronary artery, following a plaque rupture [28]. We also found a positive correlation between Galectin 3 binding protein values and other markers of inflammation, such as IL-1β, hsCRP, and fibrinogen. This finding is in line with the critical role played by inflammation in atherothrombosis, which can increase the risk of cardiovascular events, independently from cholesterol levels [29,30].

Further, in our study, patients with multi-vessel critic coronary artery disease (defined as the presence of >70% stenosis in at least two coronary vessels and detected by coronary angiography) had a higher median value of Galectin 3 binding protein than those with less critic coronary artery disease. Also, Xie et al. recently demonstrated that Galectin 3 binding protein levels positively correlate with the presence and extent of complex lesions [13].

Reinfarction after an acute myocardial infarction is a strong independent predictor of mortality [3]. Galectin 3 binding protein turned out to be able to predict the risk of angina/reinfarction at the 1 year follow-up in patients who were alive 30 days after the index acute myocardial infarction. This correlation remained significant even when adjusted for clinical variables, which had proven to be predictors of angina/reinfarction in a recent study by Arnold et al. [22]. The association between levels of Galectin 3 binding protein and a higher risk of developing another ischemic event in the first year following acute myocardial infarction is supported by the role of Galectin 3 binding protein in the inflammatory process, which leads to progression and destabilization of atherosclerotic plaques [13]. Our finding is consistent with the CANTOS trial, which showed that anti-inflammatory therapy with the anti-IL1β antibody, Canakinumab, was able to lower the recurrence of cardiovascular events, independently of lipid-lowering therapy [30]. As opposed to what was proven by Sugiura et al. [8], no association was found between Galectin 3 binding protein levels and mortality. This discrepancy can be due to the fact that our population was larger, and that we considered mortality for any cause rather than cardiovascular mortality only.

Galectin 3, a member of the galectin family that can be found both in many normal tissues and in different tumors, is involved in different physiological and pathophysiological conditions, such as development, immunity, neoplastic transformation, and metastatic spread [31]. Importantly, Galectin 3 has emerged as an independent predictor of all-cause mortality, cardiovascular death, and the occurrence of heart failure following acute coronary syndromes [7]. The Galectin 3 values of our patients are in line with values reported in other studies conducted on patients with cardiovascular disease [32] and are higher than those detected in healthy individuals [27]. Patients with Galectin 3 levels higher than the median value were significantly older and had significantly higher white blood cell counts, when compared with patients with Galectin 3 levels under the median value, as observed by Tsai et al. [32]. Galectin 3 was an independent predictor of all-cause mortality, while it was not able to predict the development of angina pectoris or myocardial infarction. The correlation between Galectin 3 levels and a higher risk of one-year all-cause death remained statistically significant, even when corrected with clinical variables associated with a higher mortality risk. This finding can be explained by the fact that, while in the first period following acute myocardial infarction Galectin 3 seems to be part of a survival mechanism to cope with the ischemic insult [10], actively contributing to the reparative processes in the infarcted area [12], in later time points, it supports the transition from acute to chronic inflammation and fibrosis. These latter two are pivotal mechanisms for the development of adverse ventricular remodeling [7,12]. Since the development of adverse ventricular remodeling after myocardial infarction is a strong predictor of mortality and heart failure [33], the association between Galectin 3 levels and left ventricular remodeling after myocardial infarction, previously proven by di Tano et al. [34], can be the basis of the higher risk of mortality observed in patients with higher levels of Galectin 3 in our study.

In the multivariable analysis, we found that Galectin 3 levels, renal function at discharge, patients’ age, and left ventricular ejection fraction were associated with 1 year mortality. These four parameters are objective and can be easily obtained. Of note, our multivariable model has proven to be comparable to the GRACE score [35] in predicting one year mortality. The GRACE score is validated in multiple databases and it includes more variables [35]. Further, we identified a Galectin 3 value of 9.82 ng/mL as discriminating between lower or increased mortality, and this value is in line with that previously identified by Tsai et al. [32]. The identification of a cut off value could be of great utility in patients’ risk stratification. 

The pathophysiological role of the tested biomarkers in the development of the different outcomes that they predict may open the road to the development of new targeted therapies. Indeed, it has been proven that mineralocorticoid receptor antagonists are able to down-regulate the expression of Galectin 3 in the infarcted myocardium of animal models and this down-regulation of Galectin 3 correlates with lower expression levels of fibrosis and inflammatory markers [36]. An interaction between the concentration of Galectin 3 and treatment with beta blockers and antialdosteronic drugs has also been observed in humans [37] so it can be hypothesized that serial measures of Gal-3 could be helpful to decide the most appropriate therapeutic schemes for each patient.

Possible limitations of our study are that we tested biomarker levels only once, within 24 h following myocardial infarction, while it would be of great interest to see if changes in biomarker levels over time could correlate with patients’ prognosis. We were not able to discriminate between cardiac and non-cardiac death. However, since patients with a short life expectancy were considered ineligible to be enrolled in our study, we can suppose that cardiac death is very likely in our population. Finally, we did not focus on left ventricular remodeling in this work, but we are now collecting follow-up echocardiographic data in order to investigate if there is any relationship between Galectin 3 levels and adverse remodeling of the left ventricle as suggested by Di Tano et al. [34]. 

## 5. Conclusions

In conclusion, in the present study, we showed that by measuring Galectin 3 and Galectin 3 binding protein in the first hours after myocardial infarction, we can better assess patients’ risk. Galectin 3 turned out to provide information about all-cause mortality, while Galectin 3 binding protein proved to be of better utility in predicting the risk or recurrent angina with the need of revascularization/hospitalization or myocardial infarction.

## Figures and Tables

**Table 1:** Baseline characteristics of all patients.

	*n* = 469
Age	67.63 (11.29)
Male sex (%)	67.8
BMI (kg/m^2^)	26.82 (4.34)
SBP at admission (mmHg)	136.02 (25.30)
DBP at admission (mmHg)	79.40 (14.47)
Heart rate at admission (bpm)	76.25 (16.82)
Cardiac arrest (%)	3
Left bundle branch block (%)	5.5
Diagnosis (%) - STEMI - NSTEMI	60.1 39.9
Killip > 1 (%)	24.4
Hypertension (%)	72.1
Diabetes mellitus (%)	24.7
Smoking (%)	45.2
Dyslipidemia (%)	59.9
Positive family history for IHD (%)	23.9
Known chronic kidney disease (%)	9.2
Peripheral artery disease (%)	7.7
Previous myocardial infarction (%)	18.3
Previous CABG (%)	3.4
Anemia at admission (%)	26.7
Total cholesterol (mg/dL)	189.43 (44.92)
LDL cholesterol (mg/dL)	118.02 (38.44)
HDL cholesterol (mg/dL)	45.26 (12.27)
Triglycerides (mg/dL)	113.5 (82–151)
TnI max (ng/mL)	12.79 (2.86–52.45)
Hb1AC (%)	6 (5.7–6.6)
Hs CRP (mg/dL)	7 (2.8–21.87)
Galectin 3 (ng/mL)	9.8 (7.77–12.77)
Galectin 3 binding protein (g/mL)	9.08 (5.77–13.54)
IL-1 β (pg/mL)	0.59 (0.42–0.97)
Na^+^ at discharge (mEq/L)	138.94 (3.35)
Hemoglobin at discharge (g/dL)	12.46 (1.67)
MDRD at discharge (mL/min)	69.37 (27.08)
GRACE score at 6 months	120.12 (31.05)
Left atrium area (cm^2^)	21.46 (5.73)
EDD_I (cm)	3 (4.79)
ESD_I (cm)	1.83 (2.46)
Interventicular septum (cm)	1.32 (1.07)
FS %	34.57 (11.3)
EDV_I (cm^2^)	47.04 (38.65–57.2)
ESV_I (cm^2^)	21.41 (16.05–28.75)
E/A	1.07 (0.77)
E/E’	11.87 (4.77)
WMSI	1.31 (1.13–1.75)
Left ventricular mass (g)	225.2 (68.26)
Left ventricular ejection fraction %	52 (47–60)
Mitral insufficiency (%) - Mild - Moderate - Severe	63.5 55.4 7.2 1.1
Therapy - PCI (%) - CABG - Medical therapy	70.6 11.1 18.2
Symptom-onset-to-balloon time (h)	2 (1 3.5)
GPIIbIIIa inhibitors (%)	10.2
Multivessel disease >70% (%)	37.5
Severe hemorrhagia (%)	1.1
Ventricular arrhythmias (%)	16.2
Supraventricular arrhythmias (%)	12.6
Brady arrhythmias (%)	7.5
Therapy at discharge (%) - ACE-I/ARB - Beta blockers - Digital - Amiodarone - Antialdosteronic agents - Loop diuretics - Aspirin - P2T12 inhibitors ⚬ Clopidogrel ⚬ Prasugrel ⚬ Ticagrelor - Statins - Oral antidiabetics - Insulin - Warfarin	75.1 77.8 1.1 7.9 11.1 24.1 93.2 40.7 26.7 19.2 89.3 15.4 9.8 8.3
NYHA class at discharge (%) - NYHA 1 - NYHA 2 - NYHA 3	88.5 8.2 3.2

BMI: body Mass Index; SBP: systolic blood pressure; DBP: diastolic blood pressure; PCI: percutaneous coronary intervention, CABG: coronary artery bypass graft; MDRD: modification of diet in renal disease; CPR: C-reactive protein; EDD: end-diastolic diameter; ESD: end-systolic diameter; WMSI: wall motion score index; FS: fractional shortening; EDV: end-diastolic volume; ESV: end-systolic volume; ARB: angiotensin receptor blockers; NYHA: New York Heart Association.

**Table 2:** (a) Main characteristics of patients stratified by the end-point recurrent angina with need of rehospitalization/revascularization or myocardial infarction. (b) Main characteristics of patients stratified by the end-point mortality.

**(a)**				
	**Final Study Cohort** ***n* = 455**	**Angina/Reinfarction** ***n* = 41 (9%)**	**No Angina/Reinfarction** ***n* = 408 (91%)**	***p*** **Value**
**Diagnosis (%)** - STEMI - NSTEMI	60 40	36.6 63.4	62.3 37.7	**0.002**
**Treatment:** - PCI - CABG - Medical treatment	71.4 11.2 17.4	73.2 4.9 22	64.8 10.8 15.4	0.8
**LVEF (%)**	54 (47–60)	57 (49–61)	54 (47–60)	0.14
**TnI max (ng/mL)**	12.5 (2.7–52)	5.78 (1.6–17.2)	13.9 (2.98–65)	**0.014**
**Galectin 3 (ng/mL)**	9.8 (7.74–12.3)	10 (8.07–11.5)	9.8(7.73–12.34)	0.8
**Galectin 3 binding protein (μg/mL)**	9.07 (5.78–13.44)	9.1 (7.36–14.2)	9.06 (5.75–13.4)	0.55
**(b)**				
	**Total Population *n* = 469**	**Died** ***n* = 35 (7.5%)**	**Alive** ***n* = 434 (92.5%)**	***p*** **Value**
**Diagnosis (%)** - STEMI - NSTEMI	60.1 39.9	57.1 42.9	60.4 39.6	0.72
**Treatment (%)** - PTCA - CABG - Medical treatment	70.7 11.1 18.2	52.9 14.7 32.4	72.1 10.8 17.1	**0.047**
**LVEF (%)**	52 (47–60)	43 (39–55)	55 (48–60)	**<0.001**
**TnI max (ng/mL)**	12.8 (2.9–52.5)	18.1 (3.6–68.4)	12.3 (2.6–51)	0.36
**Galectin 3 (ng/mL)**	9.8 (7.77–12.77)	10.49 (10–15.84)	9.74 (7.7–12.2)	**<0.001**
**Galectin 3 binding protein (μg/mL)**	9.08 (5.8–13.5)	10.4 (6.3–16.2)	9.03 (5.8–13.4)	0.10

**Table 3:** (a) Independent predictors of angina/reinfarction at multivariable Cox proportional hazards regression analysis. (b) Independent Predictors of mortality at multivariable Cox proportional hazards regression analysis.

**(a)**		
**Predictors** (Ordered by *p* Value)	**Recurrent Angina with Need of Rehospitalization/Revascularization or Myocardial Infarction**	
	**HR (95% CI)**	***p*** **value**
**Male gender (vs. female)**	4.24 (2.21–8.14)	<0.001
**STEMI diagnosis (vs. NSTEMI)**	3.5 (1.7–7.04)	<0.001
**Diabetes mellitus (yes vs. no)**	2.63 (1.32–5.18)	0.005
**Galectin 3 binding protein (for 1 unit increase)**	1.04 (1.01–1.08)	0.01
**(b)**		
**Predictors** (Ordered by *p* Value)	**All-Cause Mortality**	
	**HR (95% CI)**	***p*** **Value**
Age (for 1 y increase)	1.08 (1.04–1.12)	<0.001
EF % (for 10% increase) (for 5% increase)	0.63 (0.45–0.84) 0.79 (0.68–0.92)	0.001
LnGalectin 3 (for 1 unit increase of original variable)	3.5 (1.54–7.5) 1.12	0.002
MDRD at discharge (for 10% increase) (for 5% increase)	0.88 (0.75–1.03) 0.94 (0.86–1.01)	0.09

## Supplementary Materials

The supplementary materials are available online at https://www.mdpi.com/2077-0383/8/5/570/s1.
