# Supplementary material for: Galectin 3 and Galectin 3 Binding Protein Improve the Risk Stratification after Myocardial Infarction
Source: J Clin Med. 2019 Apr 26;8(5):570. doi: 10.3390/jcm8050570 (PMC6571589; doi:10.3390/jcm8050570)
Supplement: Supplementary file 1 [file jcm-08-00570-s001.zip › Suppl tables and figures/Supplementary Table 1_rev.docx]

**Supplementary Table 1**

Baseline characteristics of patients alive after one month after AMI

|  | n=455 |
| --- | --- |
| **Age** | 67.26(11.15) |
| **Male sex (%)** | 67.7 |
| **BMI (kg/m^2^)** | 26.82 (4.34) |
| **SBP at admission (mmHg)** | 136.23 (14.41) |
| **DBP at admission (mmHg)** | 79.74 (14.41) |
| **Heart rate at admission (bpm)** | 76.13 (16.65) |
| **Cardiac arrest (%)** | 2.9 |
| **Left bundle branch block (%)** | 5.7 |
| **Diagnosis** (%)   - STEMI - NSTEMI | 60  40 |
| **Killip >1 (%)** | 22.9 |
| **Hypertension (%)** | 72.1 |
| **Diabetes mellitus (%)** | 24.8 |
| **Smoking (%)** | 46.2 |
| **Dyslipidemia (%)** | 60.4 |
| **Positive family history for IHD (%)** | 24.2 |
| **Known chronic kidney disease (%)** | 8.6 |
| **Peripheral artery disease (%)** | 7.7 |
| **Previous myocardial infarction (%)** | 18.5 |
| **Previous CABG (%)** | 3.5 |
| **Anemia at admission (%)** | 25.5 |
| **Total cholesterol (mg/dL)** | 189.73 (44.32) |
| **LDL cholesterol (mg/dL)** | 118.35 (38.151) |
| **HDL cholesterol (mg/dL)** | 45.22 (12.26) |
| **Triglycerides (mg/dL)** | 114 (82.25-151) |
| **TnI max (ng/mL)** | 12.5 (2.66-52) |
| **Hb1AC (%)** | 6 (5.7-6.6) |
| **Hs CRP (mg/dL)** | 6.8 (2.8-20.37) |
| **Galectin 3 (ng/mL)** | 9.8 (7.74-12.3) |
| **Galectin 3 binding protein (**μg/mL**)** | 9.07 (5.78-13.44) |
| **IL-1 β (pg/ml)** | 0.59 (0.42-0.96) |
| **Na+ at discharge (mEq/L)** | 138.96 (2.98) |
| **Hemoglobin at discharge (g/dL)** | 12.5 (1.66) |
| **MDRD at discharge (mL/min)** | 70.18 (26.72) |
| **GRACE score at 6 months** | 118.59 (29.71) |
| **Left atrium area (cm^2^)** | 21.40 (5.69) |
| **EDD_I (cm)** | 3 (4.83) |
| **ESD_I (cm)** | 1.83 (2.48) |
| **Interventicular septum (cm)** | 1.33 (1.09) |
| **FS %** | 34.7 (11.19) |
| **EDV_I (cm2)** | 47.14 (38.69-56.92) |
| **ESV_I (cm2)** | 22.03 (16.75-29.14) |
| **E/A** | 1.07 (11.19) |
| **E/E’** | 11.87 (4.7) |
| **WMSI** | 1.31 (1.13-1.69) |
| **Left ventricular mass (g)** | 228.1 (71.48) |
| **Left ventricular ejection fraction %** | 54 (45-60) |
| **Mitral insufficiency (%)**   - Mild - Moderate - Severe | 64.8  57.3  7.3  0.7 |
| **Therapy**   - PCI (%) - CABG - Medical therapy | 71.4  11.2  17.4 |
| **Symptom-onset-to-balloon time (h)** | 2 (1 3.5) |
| **GPIIbIIIa inhibitors (%)** | 13.8 |
| **Multivessel disease >70% (%)** | 37.4 |
| **Severe hemorrhagia (%)** | 0.9 |
| **Ventricular arrhythmias (%)** | 16.2 |
| **Supraventricular arrhythmias (%)** | 12.5 |
| **Brady arrhythmias (%)** | 7.3 |
| **Therapy at discharge** (%)   - ACE-I/ARB - Beta blockers - Digital - Amiodarone - Antialdosteronic agents - Loop diuretics - Aspirin - P2T12 inhibitors   - Clopidogrel   - Prasugrel   - Ticagrelor - Statins - Oral antidiabetics - Insulin - Warfarin | 76.5  79.1  1.1  7.5  10.5  22.9  94.3  40.7  27.5  29.8  91.4  15.6  9.9  8.1 |
| **NYHA class at discharge**  (%)   - NYHA 1 - NYHA 2 - NYHA 3 | 89.4  7.5  3.1 |

**Legend:** BMI: Body Mass Index; SBP: systolic blood pressure; DBP: diastolic blood pressure; PCI: percutaneous coronary intervention, CABG: coronary artery bypass graft; MDRD: modification of diet in renal disease; CPR: C-reactive protein; EDD end-diastolic diameter; ESD end-systolic diameter; WMSI: wall motion score index; FS: fractional shortening; EDV: end-diastolic volume; ESV: end-systolic volume; ARB: angiotensin receptor blockers; NYHA: New York Heart Association.
