# Supplementary material for: Galectin 3 and Galectin 3 Binding Protein Improve the Risk Stratification after Myocardial Infarction
Source: J Clin Med. 2019 Apr 26;8(5):570. doi: 10.3390/jcm8050570 (PMC6571589; doi:10.3390/jcm8050570)
Supplement: Supplementary file 1 [file jcm-08-00570-s001.zip › Suppl tables and figures/Supplementary Table 2_rev.docx]

**Supplementary Table 2**. Predictors of angina/reinfaction at univariate Cox proportional hazards regression analysis

|  | **p** | HR(95%CI) |
| --- | --- | --- |
| **Gender** | <0.001 | 0.33 (0.17-0.61) |
| **Age** | 0.13 |  |
| **BMI** | 0.64 |  |
| **SBP at admission** | 0.32 |  |
| **DBP at admission** | 0.15 |  |
| **Heart rate at admission** | 0.4 |  |
| **Rhythm** | 0.77 |  |
| **LBBB** | 0.23 |  |
| **STEMI Diagnosis** | 0.001 | 0.34 (0.17-0.65) |
| **Diabetes mellitus** | 0.009 | 2.29 (1.32-4.27) |
| **Known chronic kidney disease** | 0.18 |  |
| **Previous MI/PTCA/CABG** | 0.023 | 2.15 (1.11-4.18) |
| **Killip class** | 0.14 |  |
| **Troponin I max** | 0.035 | 0.99 (0.98-0.99) |
| **EDD_I** | 0.6 |  |
| **ESD_I** | 0.67 |  |
| **EDV_I** | 0.5 |  |
| **ESV_I** | 0.17 |  |
| **LVEF** | 0.16 |  |
| **Mitral insufficiency** | 0.26 |  |
| **Multivessel disease** | 0.7 |  |
| **Sodium at discharge** | 0.3 |  |
| **Hb at discharge** | <0.001 | 0.72 (0.61-0.85) |
| **MDRD** | 0.79 |  |
| **ACEi at discharge** | 0.3 |  |
| **Beta Blockers at discharge** | 0.7 |  |
| **Antialdosteronic drugs at discharge** | 0.2 |  |
| **Loop diuretics at discharge** | 0.005 | 2.41 (1.29-4.5) |
| **Statins at discharge** | 0.96 |  |
| **Oral antidiabetics at discharge** | 0.06 |  |
| **Insulin at discharge** | 0.24 |  |
| **NYHA class at discharge** | 0.065 |  |
| **Il-1 β** | 0.5 |  |
| **CRP** | 0.1 |  |
| **Gal3bp** | 0.011 | 1.04 (1.01-1.078) |
| **LnGal3** | 0.82 |  |
