# Supplementary material for: Galectin 3 and Galectin 3 Binding Protein Improve the Risk Stratification after Myocardial Infarction
Source: J Clin Med. 2019 Apr 26;8(5):570. doi: 10.3390/jcm8050570 (PMC6571589; doi:10.3390/jcm8050570)
Supplement: Supplementary file 1 [file jcm-08-00570-s001.zip › Suppl tables and figures/Supplementary Table 3_rev.docx]

**Supplementary Table 3.** Independent Predictors of mortality at univariate Cox proportional hazards regression analysis

|  | **p** | HR(95%CI) |
| --- | --- | --- |
| **Gender** | 0.1 |  |
| **Age** | <0.001 | 1.1 (1.06-1.15) |
| **BMI** | 0.15 |  |
| **SBP at admission** | 0.5 |  |
| **DBP at admission** | 0.003 | 0.96 (0.94-0.99) |
| **Heart rate at admission** | 0.25 |  |
| **Rhythm** | 0.04 | 2.4 (1.04-5.82) |
| **LBBB** | 0.13 |  |
| **STEMI Diagnosis** | 0.7 |  |
| **Diabetes mellitus** | 0.18 |  |
| **Known chronic kidney disease** | <0.001 | 1.6 (2.35-9.8) |
| **Previous MI/PTCA/CABG** | 0.47 |  |
| **Killip class** | <0.001 | 2.49 (1.84-3.4) |
| **TnI max** | 0.24 |  |
| **EDD_I** | 0.82 |  |
| **ESD_I** | 0.93 |  |
| **EDV_I** | <0.001 | 1.024 (1.01-1.04) |
| **ESV_I** | <0.001 | 1.03 (1.02-1.04) |
| **LVEF** | <0.001 | 0.93 (0.91-0.96) |
| **Mitral insufficiency** | 0.040 | 1.44 (1.04-5.74) |
| **Multivessel disease** | 0.014 | 2.35 (1.19-4.67) |
| **Sodium at discharge** | 0.5 |  |
| **MDRD** | <0.001 | 0.975 (0.96-0.99) |
| **ACEi at discharge** | 0.006 | 0.4 (0.18-0.74) |
| **Beta Blockers at discharge** | 0.1 |  |
| **Antialdosterone treatment at discharge** | 0.045 | 2.36 (1.01-5.5) |
| **Loop diuretics at discharge** | <0.001 | 9.6 (4.3-21.58) |
| **Statins at discharge** | <0.001 | 0.2 (0.09-0.39) |
| **Oral antidiabetics at discharge** | 0.67 |  |
| **Insulina at discharge** | 0.015 | 2.84 (1.22-6.6) |
| **NYHA class at discharge** | <0.001 | 3.52 (2.61-4.76) |
| **Il-1 β** | 0.06 |  |
| **CRP** | 0.013 | 1.007 (1.002-1.013) |
| **Gal3bp** | 0.326 |  |
| **LnGal3** | <0.001 | 4.25 (2.12-8.5) |
