# Supplementary figures and images for: Galectin 3 and Galectin 3 Binding Protein Improve the Risk Stratification after Myocardial Infarction
Source: J Clin Med. 2019 Apr 26;8(5):570. doi: 10.3390/jcm8050570 (PMC6571589; doi:10.3390/jcm8050570)

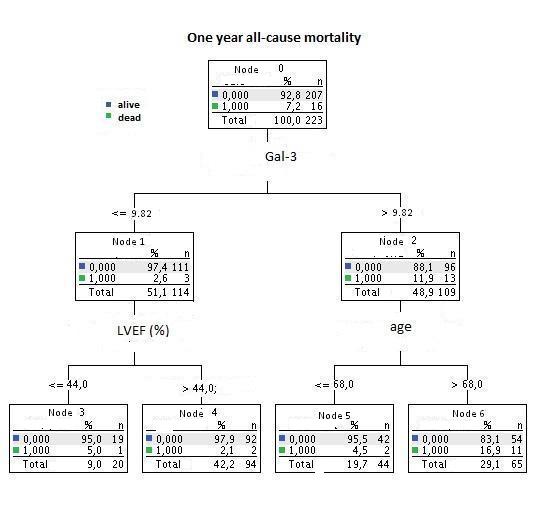

Supplement: Supplementary file 1 [file jcm-08-00570-s001.zip › Suppl tables and figures/Supplementary Figure 1.jpg]
